# Erratum: Novel bio-inspired soft actuators for upper-limb exoskeletons: design, fabrication and feasibility study

**DOI:** 10.3389/frobt.2024.1517037

**Published:** 2024-11-13

**Authors:** Frontiers Production Office

**Affiliations:** Frontiers Media SA, Lausanne, Switzerland

**Keywords:** index terms-pneumatic soft actuators, bio-inspired design, analytical modeling, wearable devices, exoskeleton

Due to a production error, there was a mistake in Figures 3–9 as published. The images were inserted in the incorrect order and did not match the respective captions. The corrected Figures 3–9 appear below.

**FIGURE 3:**
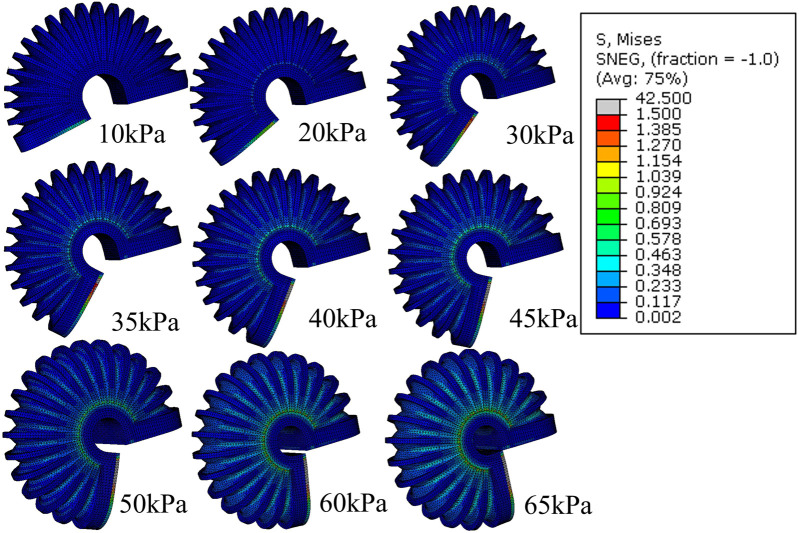
FEM simulation of the stress distribution of LISPER with a pressure range from 10 kPa to 65 kPA. Note: The c-shaped brace is hidden on the image for clear demonstration.

**FIGURE 4:**
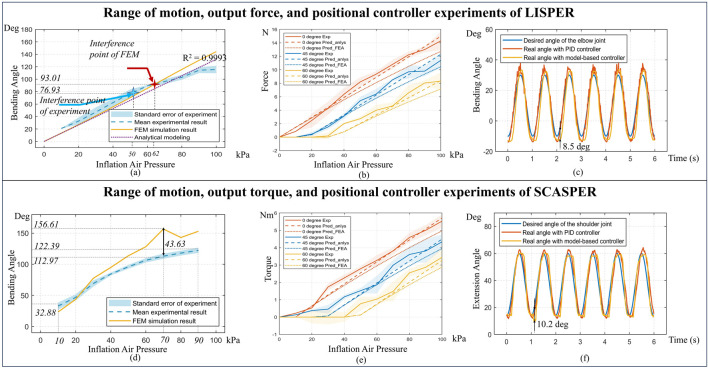
**(A, D)** Comparison between modelings and experimental bending angle for LISPER and SCASPER, respectively. **(B, E)** Comparison between analytical model-based prediction, FEA, and experiment on Pressure vs. Force and Pressure vs. Torque of LISPER and SCASPER under different fixed angles. **(C, F)** Comparison between the PID model-free controller and the model-based position controller applied to the elbow and shoulder, respectively.

**FIGURE 5:**
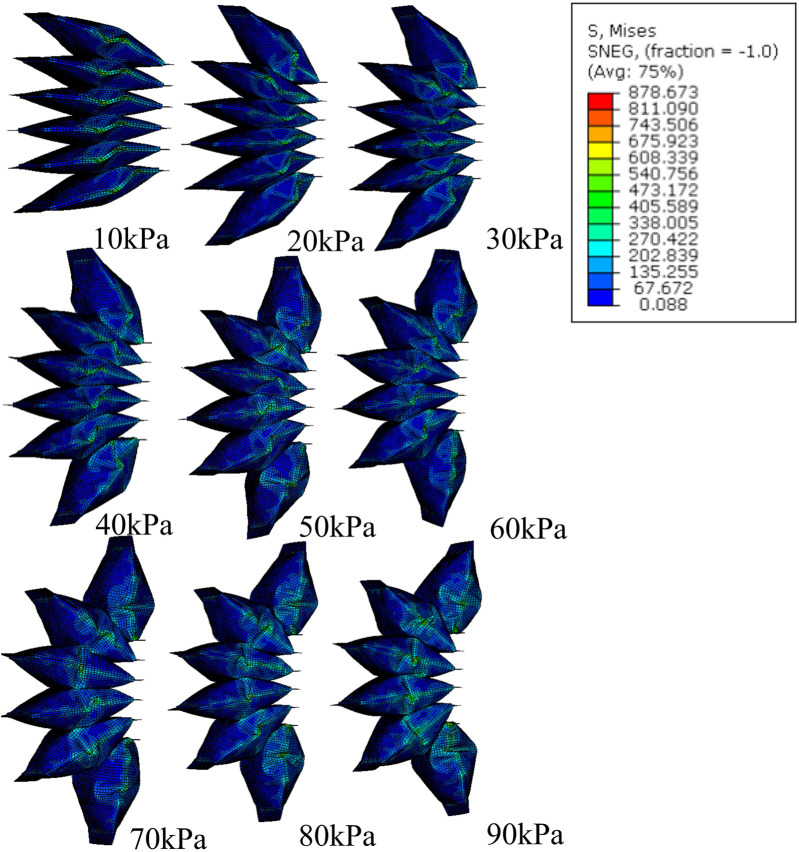
FEM simulation of stress distribution of SCASPER with pressure range from 10 kPa to 90 kPa.

**FIGURE 6:**
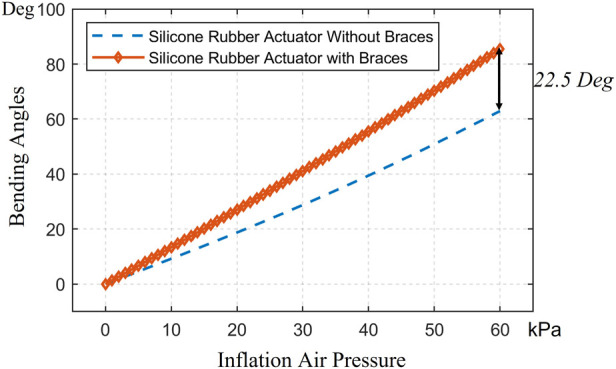
The comparison in the simulation of LISPER with and without c-shaped braces.

**FIGURE 7:**
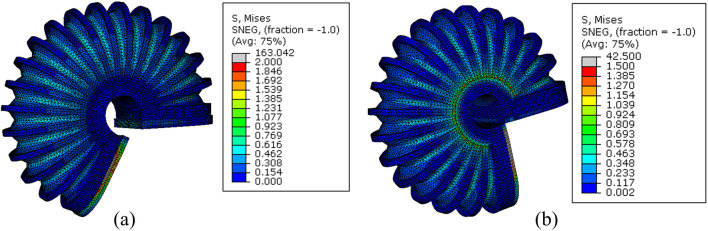
The comparison of LISPER actuator with or without braces at 65 kPa pressure input. Figure **(A)** is LISPER without braces and Figure **(B)** is with braces.

**FIGURE 8:**
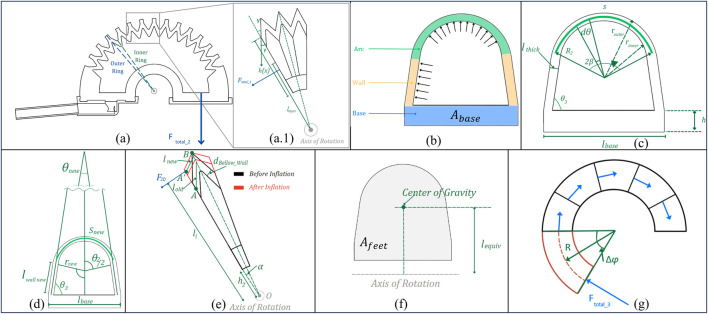
Geometric diagram of LISPER. **(A)** The sectional view of the silicone rubber body. The outer ring section is constrained by PLA rings, the inner ring is the smallest contour of each bellow segment. **(A1)** The zoomed-in view of three pieces of bellow segments. **(B)** The labeling of three sections of small ring, arc, wall, and base. **(C)** Dimension labeling of the inner ring before inflation. **(D)** Dimension labeling of the inner ring after inflation. **(F)** Equivalent center of gravity and equivalent moment of arm. **(G)** Side view of the base section when it is bent.

**FIGURE 9:**
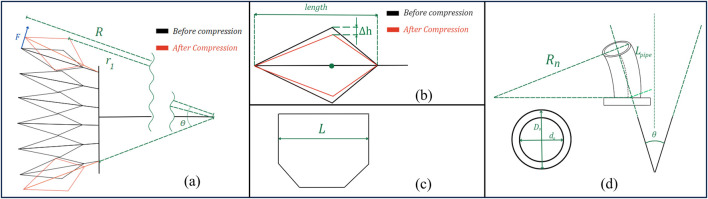
Geometric diagram of SCASPER. **(A)** The geometric labeling of SCASPER before and after compressed. F is the force output r1 is the moment of the arm from the contact point between the airbags to the center of rotation. **(B)** The sectional view of one airbag before and after compression from the environment. **(C)** The width of each airbag from the top view. **(D)** The geometric labeling of the PU pipe when they are bent.

The publisher apologizes for this mistake. The original version of this article has been updated.

