# Supplementary material for: Novel bio-inspired soft actuators for upper-limb exoskeletons: design, fabrication and feasibility study
Source: Front Robot AI. 2024 Oct 16;11:1451231. doi: 10.3389/frobt.2024.1451231 (PMC11521781; doi:10.3389/frobt.2024.1451231)
Supplement: Supplementary file 1 [file Presentation1.pdf]

Supplementary Material

0.1 Analytical Model-based Position Controller Design

Utilizing the quasi-static models of LISPER and SCASPER, we developed a model-based controller for both systems (Fig. S1(a)). By applying the model-based controllers, we enabled adaptive adjustment to varying loads on the actuators, which is a crucial aspect in clinical settings. This adaptability is essential due to the variability in limb weight and the effort exerted by patients, which can differ based on individual health conditions and inherent physiological differences. The framework was formed by the feed-forward zero torque inverse quasi-static model, where we assumed the external load to the actuators is zero. This model converts from the desired angle to the $P\_desired1$ input. Considering there are unknown loads from the environment, the real angle would differ from the desired angle. Therefore, we applied a PID controller to add the desired force to compensate for the error angle. However, since forces were already applied to the environment by the actuator, we introduced a forward quasi-static model as the force estimator to estimate the force applied by the actuator in real-time. The summation of $F\_estimated$ and $F\_desired$ are placed in the inverse quasi-static model, generating another desired pressure $P\_desired2$. The $\Delta P = P\_desired2 - P\_current$ were then summed with $P\_desired1$ and given to the actuators.

In the experimental section, the desired angle was given as a sinewave in the system. Furthermore, in the video attached, we provide the full range motion of both the actuators and their synchronized motion.

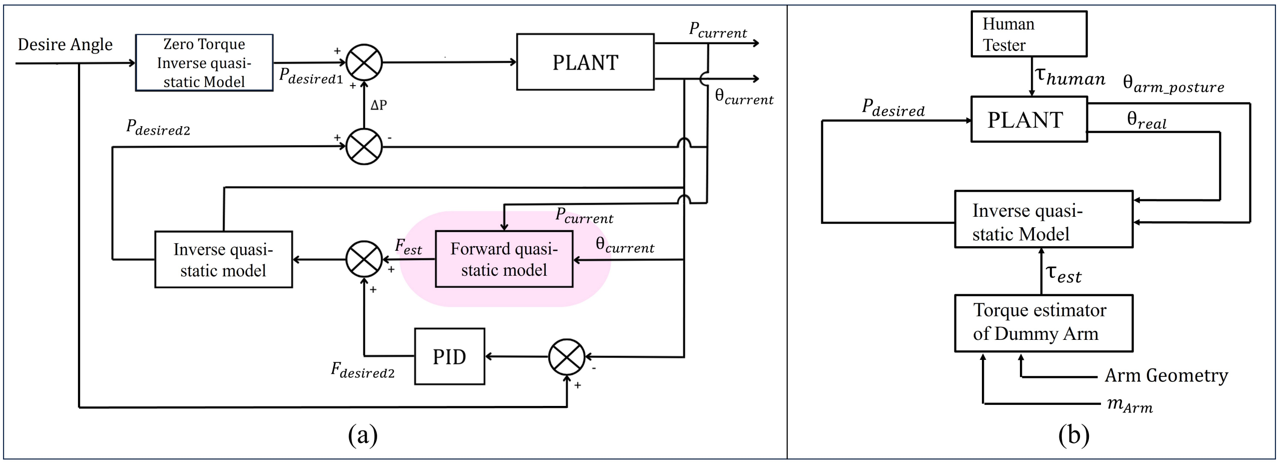

Figure S1. (a) Schematic diagram of quasi-static model-based position controller. (b) Schematic diagram of gravity compensation controller

0.2 Model-based Gravity Compensation Controller Design

Gravity compensation controllers are seldom used in soft robot-based exoskeletons. In this work, we introduce such a controller designed to maintain the 2-DOF robot arm in its current position as dictated by the designers' motion settings (Fig. S1(b)). The controller operates on the premise that both the center of mass and the mass of the human dummy are known. These parameters were inputted into the inverse quasi-static model to calculate the necessary force. This force, determined by the dummy arm's specifications, the real bending angle (θ_real), and the arm's current posture ($\theta\_Arm\_posture$), was used to compute the

required pressure (P_desire). During the experimental phase, human testers manipulated the joints of the 2-DOF human dummy arm into three distinct positions to evaluate the efficacy of this gravity compensation controller. The results of these tests are demonstrated in the accompanying video.

In the experimental sections, the human testers moved the joints of the 2-DOF human dummy arm to three different positions to test the performance of this gravity compensation controller. The results are in the attached video.
